# A Systematic Characterization Approach for Vacuum Bag Only Prepregs towards an Accurate Process Design

**DOI:** 10.3390/ma15020451

**Published:** 2022-01-07

**Authors:** Muhammed H. Arikan, Fatih Eroglu, Volkan Eskizeybek, Emine Feyza Sukur, Mehmet Yildiz, Hatice S. Sas

**Affiliations:** 1 Integrated Manufacturing Technologies Research and Application Center, Sabanci University, Tuzla, Istanbul 34956, Turkey; harikan@sabanciuniv.edu (M.H.A.); fatiheroglu@sabanciuniv.edu (F.E.); efeyza.sukur@samsun.edu.tr (E.F.S.); mehmet.yildiz@sabanciuniv.edu (M.Y.); 2 Department of Manufacturing Engineering, Faculty of Engineering and Natural Sciences, Sabanci University, Tuzla, Istanbul 34956, Turkey; 3 Department of Materials Science and Engineering, Canakkale Onsekiz Mart University, Canakkale 17100, Turkey; veskizeybek@comu.edu.tr; 4 Department of Mechanical Engineering, Samsun University, Ondokuzmayıs, Samsun 55420, Turkey

**Keywords:** prepreg, resin rheology, thermal properties, process modeling

## Abstract

Aerospace-grade composite parts can be manufactured using Vacuum Bag Only prepregs through an accurate process design. Quality in the desired part can be realized by following process modeling, process optimization, and validation, which strongly depend on a primary and systematic material characterization methodology of the prepreg system and material constitutive behavior. The present study introduces a systematic characterization approach of a Vacuum Bag Only prepreg by covering the relevant material properties in an integrated manner with the process mechanisms of fluid flow, consolidation, and heat transfer. The characterization recipe is practiced under the categories of (i) resin system, (ii) fiber architecture, and (iii) thermal behavior. First, empirical models are successively developed for the cure-kinetics, glass transition temperature, and viscosity for the resin system. Then, the fiber architecture of the uncured prepreg system is identified with X-ray tomography to obtain the air permeability. Finally, the thermal characteristics of the prepreg and its constituents are experimentally characterized by adopting a novel specimen preparation technique for the specific heat capacity and thermal conductivity. Thus, this systematic approach is designed to provide the material data to process modeling with the motivation of a robust and integrated Vacuum Bag Only process design.

## 1. Introduction

Carbon fiber-reinforced laminated composite materials have drawn significant attention from the aerospace industry in recent decades due to their high rigidity, high strength/weight ratio, and relatively high endurance to environmental factors. High-quality composite materials that can satisfy the stringent requirements of aerospace standard composites are conventionally manufactured using an autoclave process at high pressure and temperature [1]. However, the utilization of autoclaves possesses numerous disadvantages, such as high capital investment and operation cost, low energy efficiency, long process times, and constraints in the part size [2]. The motivation for manufacturing larger structural aerospace-grade composite components at lower costs without compromising the quality of the part has led to the development of next-generation materials and manufacturing processes. Accordingly, the out-of-autoclave (OoA) processes have been introduced and attracted widespread acceptance over the last decade due to their abilities to deliver composites without the need for autoclaves [2]. Subsequently, vacuum-bag-only (VBO) prepregs are explicitly developed for OoA processes, whereby high-performance primary composite structures can be manufactured through an oven-curing process with a required part quality typically achievable with autoclave processes [3]. The advantages of VBO compared with autoclave processing are the lower capital investment, the elimination of size constraints (larger parts) and the need for expensive nitrogen gas, and enabling higher energy efficiencies [4]. On the other hand, composite parts manufactured with VBO processing suspiciously include a high amount of voids caused by trapped air bubbles, which degrade the mechanical performance of the parts [5]. Unlike the autoclave processes, the maximum consolidation pressure applied during the VBO prepreg processing is the atmospheric pressure. Considering that the fiber bed carries a fair amount of this pressure, the remaining consolidation pressure on the resin may not be sufficient to discharge or suppress voids [6]. It is, therefore, critical to developing a practical methodology to migrate air bubbles, evaporated moisture, or other volatile substances towards the vacuum outlet port before gelation of the resin to produce low-void (<1 vol%) composite materials.

VBO prepregs have dry and relatively permeable air channels (engineered vacuum channels or EVaCs) that allow air removal when the vacuum is applied. The resin is progressively impregnated into these channels during the process so that it is evenly distributed and carries a low amount of voids [6]. During impregnation, the resin flow dynamics directly govern the void content in the final product, depending on the removal efficacy of the trapped air within the part [7]. Therefore, a comprehensive understanding of material constitutive behaviors and resin flow dynamics is required to obtain void-free composite structures produced with VBO processes [3].

The VBO process, in general, can be divided into three separate but interdependent components: porous media flow, heat transfer, and consolidation mechanisms [2,8,9]. The flow in porous media exists due to the resin flow between fibers. Dry fibers are expected to be impregnated with the resin while enabling air discharge. Centea and Hubert [6] observed resin impregnation in various stages of the VBO process by adapting the micro-CT imaging approach. Comprehensive mathematical models and experimental verifications involving the resin impregnation and bubble migration for VBO prepregs are available in the literature [7,10,11]. In addition to these studies, Gangloff et al. [12] evaluated void formations and bubble migration based on time, pressure, and temperature, among the process parameters. Heat transfer is included in the VBO process by several studies in the literature based on the modeling of cure kinetics and resin viscosity. The effect of cure kinetics on mechanical performances and void formation, particularly in resin-rich regions, has been investigated by various studies [8,9,13,14,15]. Kratz et al. [9] characterized the two VBO prepreg systems regarding their cure-dependent properties, cure kinetics, viscosity, and glass transition temperature following the standardized methods outlined by Khoun et al. [8]. The kinetics model’s role in predicting temperature evolution was investigated to clarify the exothermic heat generated during the curing process in thick composite parts. However, in the literature, the temperature values changing with the effect of cure kinetics were not included in the mathematical modeling, and the instantaneous value of the temperature during the process was not accurately characterized. This deficiency leads to inaccuracies in the viscosity, which also depends upon the temperature. There are also studies in the literature on the coupled effects of impregnation and cure behavior. Centea and Hubert [16] analyzed the resin impregnation with various models, including the cure kinetics and resin viscosity. They investigated the effects of the fiber architecture, temperature profile during the curing, and the initial degree of cure of the resin system through parametric studies. Additionally, they incorporated several characterization studies to develop a model for the fiber architecture. Moreover, the impact of the initial degree of cure on the degree of impregnation of the resin system, which changes with the out-times of the prepregs at room temperature prior to curing, were studied by neglecting other process parameters and its effects on void formation were reported [17]. Furthermore, heat transfer is driven by two other thermal properties: specific heat capacity and thermal conductivity. Specific heat can be expressed as the amount of heat that the material absorbs 1 °C temperature per 1 g mass, and it is usually a function of temperature. Dynamic Scanning Calorimeter (DSC) is one of the commonly preferred methods used to measure the heat capacity of materials [18]. In the literature, specific heat capacity was considered a single input to the models, and its evolution during the curing was not thoroughly characterized [17,19,20]. However, Kalogiannakis et al. [20] investigated the specific heat capacity behavior of carbon/epoxy and glass/epoxy cross-ply laminates with a Modulated Temperature Differential Scanning Calorimetry (MTDSC). They revealed that heat capacity was almost doubled between pre- and post-glass transition stages, and therefore, the heat capacity of the composites is strongly dependent on the temperature. On the other hand, thermal conductivity is another influential parameter for heat transfer since it measures a material’s capability to transfer heat. Carbon fiber-reinforced composite materials with a unidirectional fiber orientation demonstrate different thermal conductivities in the in-plane and through-thickness directions. Hence, the thermal conductivity of the resin and fiber components of the prepreg could be investigated individually by certain thermal conductivity models [18]. In addition to the effect of geometrical disposition and fiber/resin fraction, thermal conductivity is a temperature-dependent property, which means that the material can demonstrate different thermal conductivity behaviors in different temperature conditions. According to the literature, although thermal conductivity is a temperature-dependent property, it varies slightly in a limited temperature range [19,20]. Therefore, it is preferable to conduct the experiments in a wide temperature range to observe the noticeable differences in the thermal conductivity of the constituents.

Another mechanism that needs to be included in the modeling is consolidation. Consolidation is studied in the literature as the resin flow within a fiber architecture. Various mathematical models and numerical analysis methods were developed for VBO prepregs [2,21] to understand the effects of the consolidation mechanism. Gangloff et al. [10] investigated the interactions between engineered air channels and consolidation, and they demonstrated the influence of the consolidation profile on void formation. Centea and Hubert [22] performed a parametric study for the consolidation profile under different pressures for the VBO process and analyzed the effect of the consolidation mechanism on the microstructure of the final product. They concluded that the effects of other process parameters need to be taken into account.

**Figure 1:**
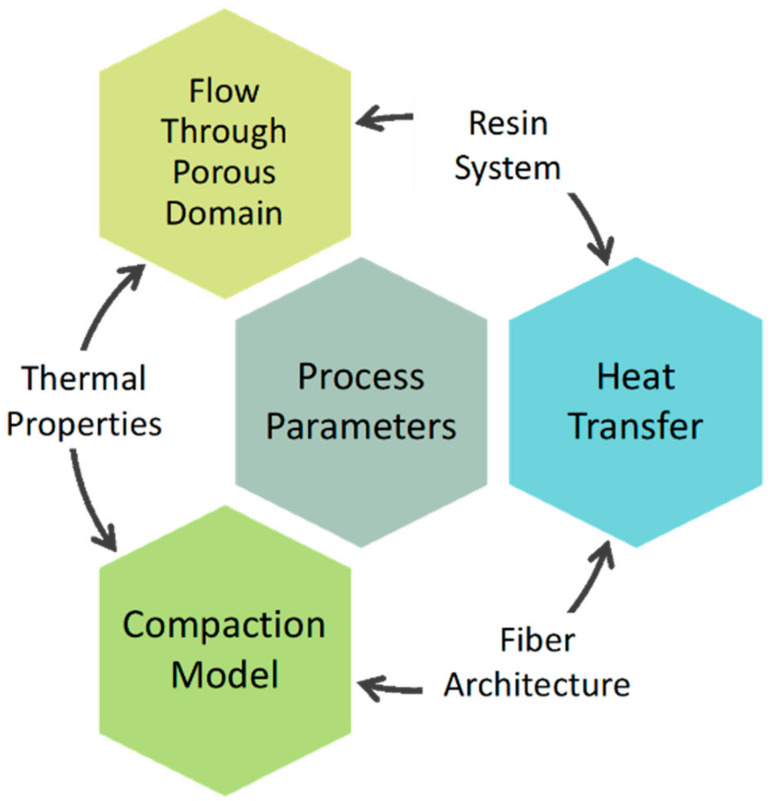
Governing physics of VBO process and integration with the prepreg material properties.

The design of the VBO process based on the fluid flow, consolidation, and heat transfer should be linked to accurate material parameters of the prepreg and its constituents to derive acceptable process parameters, as schematically shown in Figure 1. For this reason, there is a vital need for a systematic and inclusive characterization study for VBO prepreg material characterizations. Considering the studies in the frame of VBO process design with various perspectives, to the best of authors’ knowledge, there is no systematic characterization study in the literature focusing on related prepreg material properties in an integrated manner with the physics of the VBO process. This study intends to establish a methodology that systematically characterizes the VBO prepreg properties and develops constitutive behavior to strengthen the accuracy and reliability of the VBO process model. Accordingly, this approach is applied to characterize the properties of a commercial VBO prepreg system and was carried out in three steps. First, for the resin system, the cure-dependent properties are characterized regarding cure kinetics, glass transition temperature, and viscosity by semi-empirical phenomenological models. Second, the fiber architecture is investigated for the resin film, fibrous region, and void-content change and the fiber volume fraction of the prepreg system through sets of X-ray tomography scans of the uncured and cured samples. This study is followed by the numerical permeability characterization of the initial porous media, modeled through laminar flow analysis of the selected domain. Finally, the specific heat capacity and thermal conductivity of the constituents are measured by a novel experimental design. This novel integrated prepreg material characterization recipe maintains the numerical implementation with improved reliability of the process modeling and optimization towards the success of the VBO process design.

## 2. Materials and Methods

### 2.1. Materials

A KOM12 UD300 (KORDSA Global, Istanbul, Turkey) carbon fiber-reinforced (CFRP) prepreg system explicitly designed for the VBO process was used for this study. The prepreg was composed of unidirectionally aligned 12K carbon fibers with a fiber weight ratio of 300 g/m^2^ [23]. The OM12 resin system (KORDSA Global, Istanbul, Turkey) was an epoxy-based system with the suggested cure temperature of 80–130 °C. The densities of the resin and carbon fibers were 1180 and 1850 kg/m^3^, respectively. The uncured prepreg samples were kept at a temperature of −18 °C, and before the experiment, the prepreg samples were stored at 4 °C for 24 h.

### 2.2. Methodology

**Figure 2:**
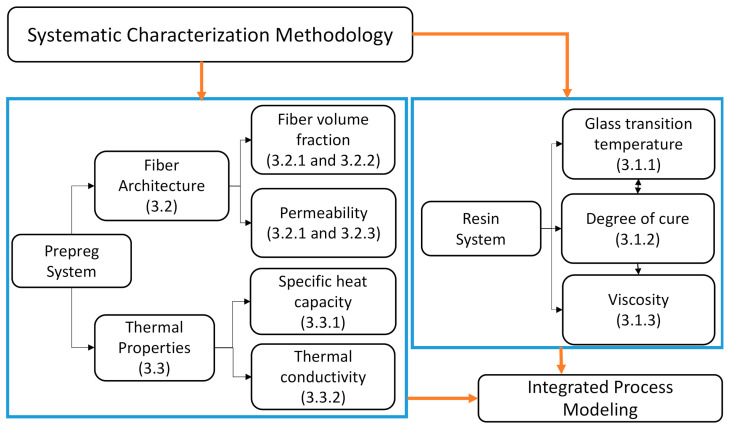
Systematic characterization road map of the prepreg material and its constituents with the numbers in parenthesis addressing the subsection with the corresponding characterization.

A robust VBO process model that enables successful aerospace-grade manufacturing requires process modeling, process optimization, and validation, built on systematic material characterization. For VBO prepregs, the physical and chemical characterizations should be conducted not only for the prepreg system but also for its constituents, including the resin system and fiber structure. Figure 2 depicts the systematic approach used to study the process-related properties of the prepreg system with the resin and the reinforcement components, individually. For the resin characterization, the main physical properties affecting the process were addressed: (1) cure kinetics (model for degree of cure (α)), (2) glass transition temperature (T_g_), and (3) viscosity (μ). These properties are dependent on a parameter: degree of cure (α). However, as shown in Figure 2, T_g_ is correlated with α, while μ is related to α. First, the cure behavior of the resin system was studied to capture the evolution of the degree of cure, α, as a function of time and temperature. Accordingly, the rheological behavior of the resin was expressed as a function of the degree of cure, temperature, and time. For the prepreg system characterization, fiber architecture, thermal behavior, and the resin system are crucial to understanding and designing the VBO process in an integrated manner. In the case of addressing the permeability, which is the leading parameter for successful air removal during the VBO process, the fiber bed architecture in the tow-scale needs to be identified, including the initial locations of the resin. Therefore, prepreg laminates with different curing stages were analyzed to investigate the porosity and permeability and to subsequently model the tow geometry of the prepreg system. Furthermore, thermal behavior was studied with thermal conductivity and specific heat characterization studies. The proposed approach is presented in the following sections with details on the systematic characterization methodology and the established material constitutive models.

## 3. Characterization Studies

As the roadmap of the systematic characterization is introduced with Figure 2, this section presents the characterization methodology for each parameter with the corresponding findings.

### 3.1. Resin System

#### 3.1.1. Cure Kinetics

The cure behavior of the thermosetting resin can be predicted using a phenomenological model combined with a series of dynamic scanning calorimetry (DSC) experiments. In this study, experimental results were used to fit the parameters to a diffusion-controlled autocatalytic equation developed by Hubert et al. [24]. They adapted a cure kinetics model (Equation (1)), formerly developed by Castro et al. [25], and combined with another model developed by Kamal and Sourour [15] to account for the reactions at low degrees of cure.

(1) $$\frac{d\alpha }{\mathrm{dt}}=K_{1}\alpha^{m_{1}}{\left(1-\alpha \right)}^{n_{1}}+\frac{K_{2}\alpha^{m_{2}}{\left(1-\alpha \right)}^{n_{2}}}{1+e^{\left(D\left(\alpha -\left(\alpha_{C0}+\alpha_{\mathrm{CT}}T\right)\right)\right)}}\;\mathrm{with}\;K_{i}=A_{i}e^{-\frac{E_{A_{i}}}{\mathrm{RT}}},\;i=1,2$$

where dα/dt is the cure rate, K_1_ and K_2_ are the Arrhenius temperature dependency as in Equation (1), I represents the primary/secondary epoxy-amine reactions, A is the pre-exponential coefficient, E_A_ is the activation energy, R is the universal gas constant, and T is the absolute temperature. In Equation (1), D is the diffusion constant; m_1_, m_2_, n_1_, n_2_, and α_C0_ are the critical degrees of cure at absolute zero temperature; and α_CT_ corresponds to the increase in the critical resin degree of cure with temperature. Mettler Toledo DSC 3+, Zurich, Switzerland was utilized to measure the heat flow in dynamic and isothermal conditions. There were four performed sets of dynamic scans with heating rates between 5 and 20 °C/min, starting from −60 up to 350 °C, to obtain the total heat of reaction of the resin system. Furthermore, isothermal dwells were performed at three different temperatures, 100, 120, and 130 °C, to determine the isothermal heat of the resin, and these dwell temperatures were selected based upon the manufacturer’s recommended cure cycle [23]. After each isotherm was completed, the sample was cooled to room temperature and heated up with a specific heating rate of up to 300 °C to determine the residual heat of the reaction. Each experiment was carried out two times to validate the reproducibility of the results.

The cure rate was obtained from the DSC data by converting the measured heat flows into cure rates following the techniques outlined by Khoun et al. [8]. The first step was to convert the heat flow values obtained from dynamic DSC scans to the total heat of the reaction. This can be simply described as the area between the heat flow and the baseline curves. The average total heat of reaction for the OM12 epoxy resin system was determined as 340 J/g with a standard deviation of 3%.

On the other hand, α was determined through the isothermal scans by comparing the isothermal (H_I_) and residual (H_R_) heats of the reaction with a fixed total (H_T_) heat of the reaction as stated in Equation (2).

(2) $$\alpha =\frac{H_{T}-H_{R}}{H_{T}}$$

The degree of cure values of the resin system for different conditions could be obtained with Equation (4), and then, it was utilized to set the baseline to find out the isotherm values as in Equation (4), which contributes to determining the experimental cure rate (dα/dt) of the reactions provided in Equation (5).

(3) $$H_{I}+H_{R}=H_{T}$$

(4) $$\frac{d\alpha }{\mathrm{dt}}=\frac{1}{H_{T}}\times \frac{\mathrm{dH}}{\mathrm{dt}}$$

Hence, the cure rate of the resin system could be expressed as a function of the degree of cure and compared with existing cure kinetics models (Equation (1)), and E_A_ was obtained from the Arrhenius equation provided below (Equation (5)):

(5) $$\frac{d\alpha }{\mathrm{dt}}=A\times e^{-\frac{E_{A}}{\mathrm{RT}}}$$

which can be rewritten as follows:

(6) $$\ln\left(\frac{d\alpha }{\mathrm{dt}}\right)=\ln\left(A\right)-\frac{E_{A}}{\mathrm{RT}}$$

From Equations (5) and (6), E_A_ was obtained by calculating the slope of ln(dα⁄dt) versus (1⁄T) at a low degree of cure (α = 0.1) and the procedure was repeated for the other activation energy value. Additionally, a linear relationship could be obtained between the ultimate degree of cure and the glass transition temperature. Therefore, α_C0_ and α_CT_ were determined as the parameters of the linear fit. Other parameters, A_1_, A_2_, D, m_1_, m_2_, n_1_, and n_2_ were determined by using a least-squares nonlinear regression curve fit between the cure rate and the degree of cure values for a complete set of experiments. All of the dynamic and isothermal trials were fitted simultaneously with the script, and one set of parameters was obtained to fit all conditions. The evolution of the resin degree of cure was precisely characterized by the cure kinetics model (Equation (1)) with the predicted parameters given in Table 1.

**Figure 3:**
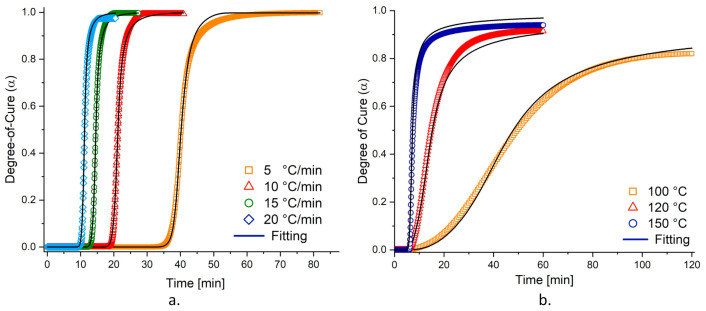
Degree of cure profiles of (**a**) dynamic heating rates and (**b**) isothermal temperature profiles. The experimental data (symbols) is compared with the model predictions (continuous lines) (For an interpretation of the references to the colors in this figure’s legend, the reader is referred to the web version of this article).

To present the fitting quality of the developed cure kinetics model, the predicted and experimental degrees of cure were compared for both dynamic and isothermal conditions in Figure 3, respectively. These results present that the cure kinetics model precisely predicts the curing evolution of the resin system for both dynamic and isothermal conditions. The model slightly deviates from the experimental degree of cure for isothermal dwells at 120 and 150 °C. However, this imperfection can be tolerated as the success of the fit is indicated by the lowest R2 value of 0.96, which belongs to the dwell of 150 °C.

#### 3.1.2. Glass Transition Temperature

The glass transition temperature (T_g_) model was developed using the residual part of the isothermal DSC experiments and fitting the experimental data to the model developed by DiBenedetto [26]. The DiBeneddetto model equation is:

(7) $$\frac{T_{g}-T_{g_{0}}}{T_{g_{\infty }}-T_{g_{0}}}=\frac{\lambda \alpha }{1-\left(1-\lambda \right)\alpha }$$

where T_g_ is a function of α and dependent on the glass transition temperature of the uncured resin $T_{g_{0}}$ and the fully cured resin $T_{g_{\infty }}$. λ is a fitting parameter in this equation, and it was predicted based on the least-squares nonlinear regression.

The methodology introduced by Kratz et al. [9] was adapted to experimentally determine the T_g_ values. The T_g_ values of the partially and fully cured resin systems were obtained with a series of DSC and dynamic mechanical analysis (DMA, Mettler Toledo DMA/SDTA 861e, Zurich, Switzerland) experiments, respectively. As explained in the Cure Kinetics section, after each isothermal dwell (100, 120, and 150 °C), samples were cooled down to the room temperature and heated up with a specific temperature rate up to 300 °C. T_g_ of the partially cured resin was taken as the midpoint in the dramatic change in the heat flow versus temperature graph during the ramp. To obtain the T_g_ of the fully cured resin, 8 plies of prepreg were stacked and cured in an oven following the manufacturer’s recommended cure cycle [23]. After the curing process, samples were tested in three-point bending mode in DMA according to ASTM D7028-07. The samples were subjected to 100 mm sinusoidal displacements at a frequency of 1 Hz, while the test chamber was heated at 3 °C/min.

**Figure 4:**
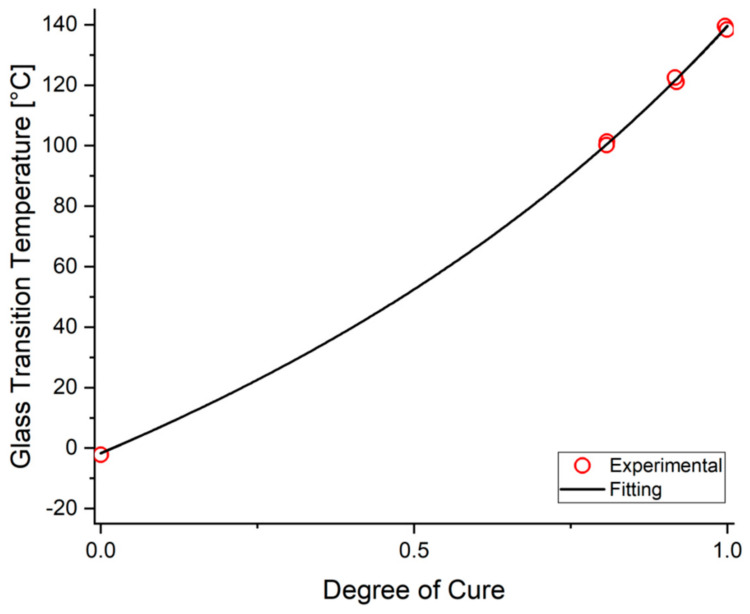
The measured and predicted glass transition temperatures for OM12.

The fitting results of the resin glass transition temperature model are demonstrated along with the experimentally determined average T_g_ values, as given in Figure 4. As seen, this model accurately captures the glass transition temperature behavior of the resin system as a function of the degree of cure. The final parameters of the glass transition model are summarized in Table 2.

#### 3.1.3. Viscosity

The viscosity of the resin system can be characterized using the semi-empirical models as a function of temperature and the degree of cure by coupling with the cure kinetics model [8,9]. The viscosity model used in this study was first developed by Khoun et al. [8]. They adapted a viscosity model that includes the gel effects, formerly developed by Castro et al. [25], to incorporate an additional Arrhenius temperature dependency and a polynomial term to describe the viscosity behavior at gelation point better. The model is as follows:

(8) $$\mu =\mu_{1}+\mu_{2}{\left(\frac{\alpha_{\mathrm{gel}}}{\alpha_{\mathrm{gel}}-\alpha }\right)}^{A+B\alpha +{C\alpha }^{2}}$$

where α is the instantaneous degree-of-cure predicted using Equation (1); α_gel_ is the degree of cure at a gelation point; A, B, and C are the numerical constants calculated using the least-squares nonlinear regression between the viscosity and the temperature; and μ_i_ is the Arrhenius temperature dependency:

(9) $$\mu_{i}=A_{\mu_{i}}\times e^{\frac{E_{\mu_{i}}}{\mathrm{RT}}}\;,\;i=1,2$$

where $E_{\mu_{1}}$ and $E_{\mu_{2}}$ are the viscosity activation energies, A_1_ and A_2_ are experimentally determined pre-exponential constants, R is the universal gas constant, and T is the absolute temperature.

The rheological behavior of the neat resin was characterized using an Anton Paar MCR 702 TwinDrive, Graz, Austria rheometer. The four sets of dynamic scans starting from 50 °C up to 160 °C with a variety of temperature ramps between 1–4 °C/min were performed for rheological characterization. The experiments were conducted in oscillatory mode at a controlled strain of 0.01% and a constant frequency of 1 Hz until the termination criteria, Loss Modulus = Storage Modulus and tan(δ) = 1 (G-crossover point), was reached. The resin specimens were placed between disposable parallel plates with a diameter of 25 mm and a thickness of 1 mm. $E_{\mu_{i}}$ and A_i_ were determined following the same approach explained in the Cure Kinetics section. The ln μ was plotted versus 1/T from room temperature until the viscosity began increasing. The slope and intercept of the linear trendline were used to determine $E_{\mu_{i}}$ and A_i_, respectively [9]. Additionally, the degree of cure at gelation point, α_gel_, was determined as 0.75 by taking the average of G-cross over the point from each dynamic [16,27]. Later, the degree of cure behavior of the resin system during the viscosity experiments was modeled by generating a script based on the cure kinetics characterization methodology described in the previous section, which incorporates Equation (1) along with the predetermined model parameters. In addition, other model constants were calculated through a script that adapts a least-squares nonlinear regression curve fit with the experimental data. All the dynamic trials were fitted simultaneously, and one set of parameters was obtained for the OM12 resin system viscosity model (Table 3).

**Figure 5:**
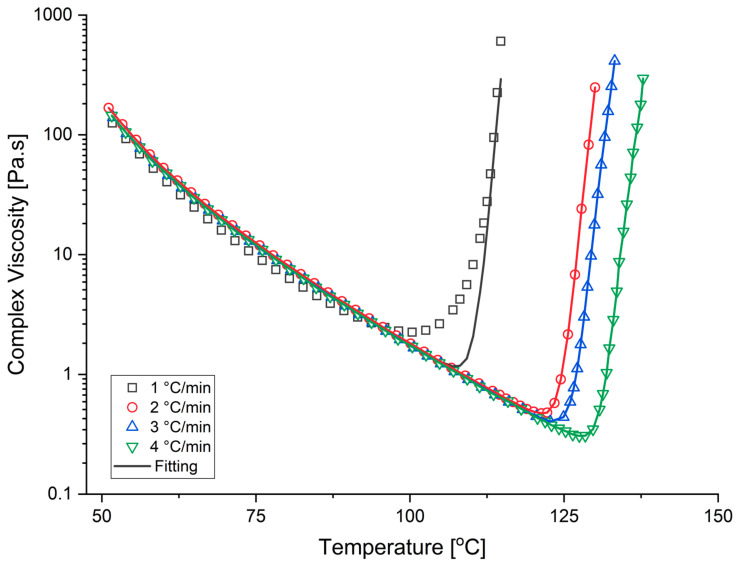
Experimental (symbols) and model predictions (continuous lines) for complex viscosity profiles under dynamic conditions (For an interpretation of the references to the colors in this figure’s legend, the reader is referred to the web version of this article.).

Figure 5 presents the measured viscosity response of the resin under a set of dynamic conditions along with the model predictions. Overall, the model exhibits a good agreement with the experimental data and accurately predicts the onset of gelation point, which is α = 0.75 for OM12 resin system. There is a slight deviation between the experimental and predicted viscosity values; the model underestimated the minimum viscosity value and viscosity at the gelation point for 1 °C/min temperature ramp. On the other hand, the viscosity model successfully captured the evolution of the resin viscosity, temperature ramp, and gelation for temperatures ramp greater than 1 °C/min. Additionally, considering the fact that the OM12 resin system is developed for cure temperatures between 80 and 130 °C, this model precisely predicts the viscosity evolution during the temperature ramp. In the viscosity characterization study, the isothermal experimental data are not adapted due to the notable deviations over the predefined isothermal dwell temperature during the experimentation. This might result from the fast cure nature of the resin type and the experimental parameters such as frequency and strain.

By means of this systematic analysis, the integrated characterization of the resin system with α, μ, and T_g_ in Figure 2 is completed.

### 3.2. Fiber Architecture

Dry fiber fabrics should be successfully impregnated with the resin via air evacuation during the consolidation and subsequent oven curing stage based upon the requirements of the VBO process. Hence, it is essential to comprehend the initial state of the fiber architecture to reveal the fiber volume fraction, which leads to the permeability characterization of the reinforcement system. In this study, prepreg samples with different curing stages were investigated using micro-CT scans and their following analyses. These sets of samples included uncured and cured prepregs to examine the first fiber architecture, void content, and fiber volume fraction along with the effects of compaction, resin flow, and subsequently curing on these parameters.

#### 3.2.1. Micro-CT Analysis

Experimental studies exhibited that, in addition to resin viscosity and cure cycle, the initial stage of the prepreg should be carefully addressed for a successful process with minimum void content. For this, accurate and reliable inspection and visualization techniques become essential from the material design viewpoint. X-ray computed tomography (Micro-CT, SkyScan 1172 Desktop, Kontich, Belgium) has been suggested as a well-adapted precise tool to reveal the pre- and post-cured microstructure of composite materials [28].

In this study, to examine the pre-and post-process properties of the reinforcement system, uncured and cured samples were prepared. In the case of preparing the uncured sample, prepreg ply with 10 × 10 mm^2^ were cut from the prepreg roll with a ZUND digital cutter (Altstätten, Switzerland). As for the cured sample, one ply with a size of 300 × 300 mm^2^ was prepared on an aluminum tool for the oven-curing process. The process cycle for the laminate was initiated with a full-vacuum hold for 10 min at 25 °C, 2 °C/min ramps to 85 °C for an hour-long isothermal dwell, and another ramp to 120 °C (final cure temperature) for another hour-long isothermal dwell followed by cooling with 2 °C/min ramp to 60 °C for a total process time of 210 min. Following the curing process, the sample with the dimensions of 10 × 10 mm^2^ was removed from the center.

For micro-CT analysis, relevant scanning parameters [6] were adjusted to acquire the optimum contrast between the carbon fiber, epoxy resin, and voids, as shown in Table 4. Micro-CT scans take approximately two hours for each sample for 360° rotation. NRecon (Skyscan) software, Kontich, Belgium was utilized to reconstruct the projections into sequences of parallel X-ray micrographs. Reconstruction settings as misalignment compensation, ring artifact reduction, and beam hardening reduction were adjusted for each set of micrographs through a series of parametric studies performed with NRecon’s fine-tuning option. The variation of the macro-void content of each sample was quantified using CTAn (Skyscan), Kontich, Belgium 3D image analysis software.

**Figure 6:**
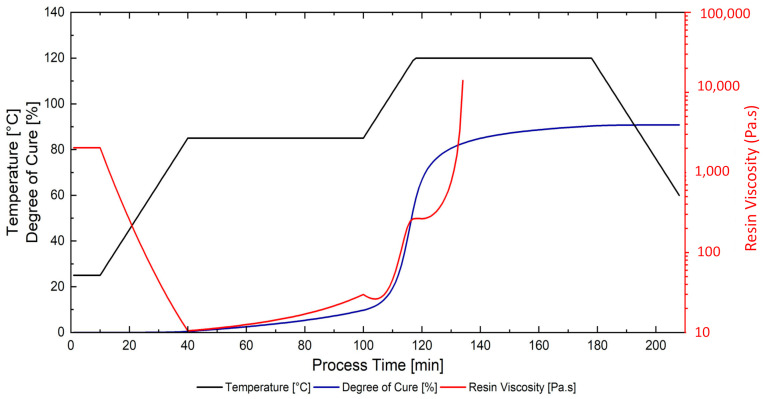
Process cycle for the cured laminates: oven temperature profile and numerically predicted resin degree of cure and viscosity.

The preparation of the scanning samples includes the application of the cure cycle temperature, the numerically predicted degree of cure, and viscosity properties of the resin system were calculated from Equations (1)–(9) and the model parameters listed in Table 1 and Table 3 with the cure cycle temperature, as it is given in Figure 6. The numerically predicted resin degree of cure could reach up to α = 0.9. In contrast, the predicted resin viscosity decreased from almost μ = 1600 Pa·s (at room temperature) to a minimum value of μ = 10 Pa·s (at the beginning of the 85 °C isotherm) throughout the cure cycle. During the first dwell (at 85 °C), the viscosity gradually increased. However, with the cure kinetics of the resin system, the viscosity ascended very quickly to the gelation point (predicted to be α = 0.75) despite the contrary effect of the temperature ramp on the viscosity.

**Figure 7:**
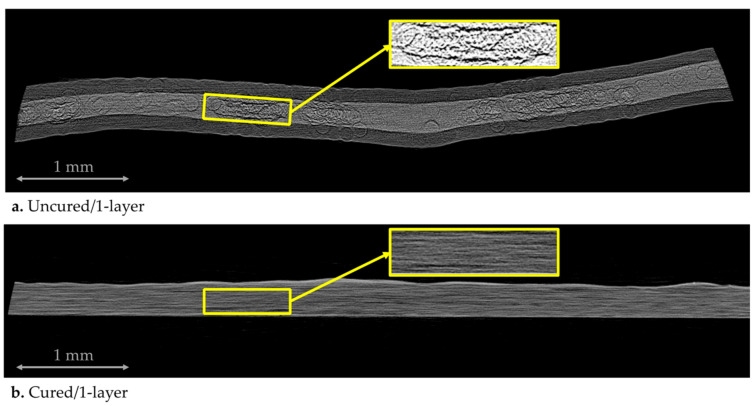
X-ray micrographs: (**a**) uncured/1-layer and (**b**) cured/1-layer. Additional inserts highlight the visible dry fiber tow areas for uncured samples and relatively dry regions for cured samples.

Figure 7 exhibits the representative micrographs for the two samples. In the micrographs, voids were denoted as black since these areas possess zero attenuation. In contrast, brighter grayscale values stand for denser, resin-rich regions. Figure 7a depicts the initial state of uncompacted and uncured prepreg ply (uncured/1-layer) in detail. Resin films were placed on top/bottom of the ply and denoted with relatively brighter grayscale value. We can assume that these resin films do not contain fibers. On the other hand, a fiber bed was presented between the resin films and depicted with the brightest grayscale value. Furthermore, as given as the inset, a mixture of solids and black voids represents the resin-rich regions surrounding the dry fiber tow areas. Additionally, large gaps are present within the dry fiber regions due to entrapped air between the dry fiber tows in the preparation process. Figure 7b presents consolidated and cured prepreg laminate (cured/1-layer). As can be seen from the insert, the cured/1-layer sample stands out as a prepreg in which a large proportion of local voids were eliminated.

#### 3.2.2. Fiber Volume Fraction Measurements

**Figure 8:**
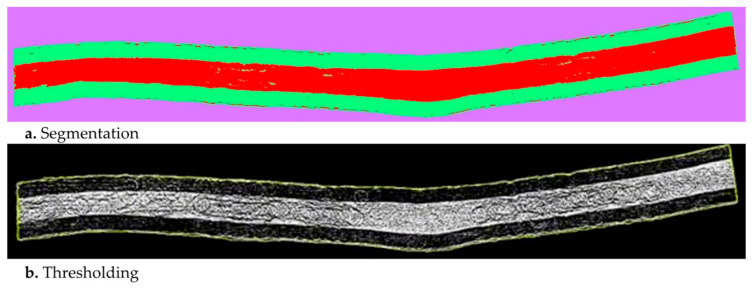
Image processing of the CT images: (**a**) region segmentation with Image J of prepreg sample representative slice for the (purple) background, (green) resin, and (red) fiber and (**b**) region of interest adapted for each slice of the prepreg sample (highlighted as yellow lines).

The fiber volume fractions were calculated from the CT images via ImageJ, Bethesda, MD, USA, an open-source image analysis software. First, the image segmentation was performed to identify the resin film region and the dry fibrous region (Figure 8a). The reconstructed CT images were imported into the software and a threshold with a higher grayscale value was chosen to separate the raw images into black (reinforcement) and white (resin and voids). Then, a series of the region of interest were manually selected from the first, intermediate, and end slices to interpolate them for the optimal region of interest per micro-slice. Following that, a 2D analysis was performed to measure the area fraction of the fibers inside the region of interest for each slice (Figure 8b), and the resultant set of fractions was averaged to obtain the fiber volume fraction of the sample.

Based on the Micro-CT analysis, the obtained data are presented in Table 5 along with their corresponding process stage. As provided for the uncured sample considering the domain as resin films and fiber bed, the fiber volume fraction was evaluated as 33.5% (Figure 8b). Additionally, the thickness was 0.5 mm, including 0.125 mm for each resin film on the top and bottom and 0.25 mm for the fiber bed. The volume fraction for the fiber bed for the uncured sample was obtained using the thickness ratios of the resin films and the fiber bed: 67% (33.5% × 2). Using the Micro-CT for the cured sample, the fiber volume fraction was calculated as 67.59% using the image processing procedure. When the results of the uncured sample were compared with the cured one, the uncured and uncompacted sample exhibited higher void contents and average ply thicknessed and lower fiber volume fraction. Subsequent to the application of consolidation and curing, void content and average ply thickness demonstrated noticeable decreases while the fiber volume fraction increased to a large extent. This highlights the significance of debulking on closing the inter-ply gaps and leading the remaining air to the EVaCs during the processing. Evidently, a significant increase in fiber volume fractions could be observed due to consolidation and curing processes for the single-layer prepreg samples (uncured/1-layer and cured/1-layer). Additionally, the Micro-CT analysis provided that the average fiber diameter is 3 μm.

Additionally, the initial fiber volume fraction was validated by Soxhlet extraction for the uncured prepreg. An automatic solvent extractor (VELP Scientifica—SER 158 Series) was used to remove the matrix material from the carbon fiber reinforcement as recommended by the ASTM C613—19 standard. By applying the rule of mixtures and knowing the densities of resin and reinforcement, the initial fiber volume fraction was calculated to be 31.4%.

#### 3.2.3. Numerical Permeability Characterization

**Figure 9:**
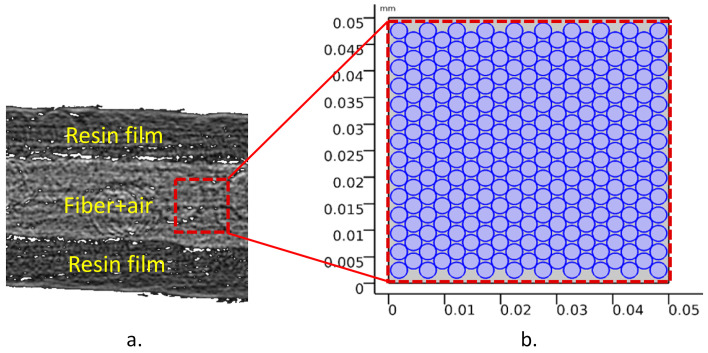
Model domain for numerical permability characterization: (**a**) CT image, (**b**) representative numerical model domain with the dry fiber bed with fibers (dark circles) and air (light areas).

The permeability values that represent the ease of resin impregnation through a dry fibrous fiber bed, playing a crucial role in void formation, should also be quantified [29]. In this study, the permeability values used to identify the flow along the cross-sectional area of the fiber bed were numerically characterized [30,31,32,33]. Utilizing the parameters provided in the previous section, fiber volume fraction of fiber bed (67%), and the average fiber diameters (3 μm) of uncured prepreg, the solution domain for the simulation of laminar viscous flow was generated, as given in Figure 9. The numerical simulation of the laminar flow by assuming the fibers as solid walls was performed with the “Creeping Flow” module of the COMSOL Multiphysics^®^ software (version 5.4, Burlington, MA, USA) with the domain and boundary conditions given in Table 6. Therefore, the numerical solution with a constant pressure difference along the top to the bottom direction (by neglecting the gravitational effect) for the square domain with dimensions of 0.05 mm × 0.05 mm provides the flow rate, q, in the top-to-bottom direction [16]. The Darcy’s Law for flow through porous media in one-dimensional form was adapted to derive the permeability value in the flow direction with the equation below [34]. In the equation, $K\;\left(m^{2}\right)$ is the permeability of the porous medium, $\mu \;\left(\mathrm{Pa}\cdot s\right)$ is the dynamic viscosity of the fluid, $q\;\left(m/s\right)$ is the flux discharge per area, and $∆P\;\left(\mathrm{Pa}/m\right)\;$ is the pressure gradient vector.

(10) $$K=\frac{\mu \times q}{∆P}$$

As the numerical simulation data of the flow rate were implemented with Equation (10), the permeability value of the airflow between fibers in this micro-scale model was calculated as 2.02 × 10^−15^ m^2^. This value could be defined as the initial air permeability.

### 3.3. Thermal Behavior

Thermal properties such as specific heat capacity and thermal conductivity of the prepreg constituents possess significant influence over the resin flow dynamics as curing is dependent upon the temperature profile during the process, and therefore, the thermal properties of the resin system should also be characterized.

#### 3.3.1. Specific Heat Capacity

The specific heat capacity of the prepreg system and the resin system itself were distinctively characterized with DSC analysis. Measurements were performed at a temperature range from −60 to 350 °C with a heating rate of 10 °C/min in dynamic conditions. As suggested in the literature [19], the DSC instrument was calibrated with a sapphire sample within the same temperature range with a similar heating rate before each test. DSC experiments were repeated three times to validate the reproducibility of the results.

The DSC apparatus measures the heat flux (dH/dt) during the temperature ramp (dT/dt), and it is usually a function of the temperature with a constant slope. Therefore, linear regression is often sufficient to predict the variation in the specific heat capacity of the resin system during the curing period (80–220 °C for OM12 resin system) [19,20,35]. Hence, linear regression was applied over a temperature range of 20–220 °C to predict the specific heat capacity behavior of the materials accurately.

Furthermore, the commonly used rule of mixture method [18] (Equation (11)) was applied to prepreg and resin system results to predict the specific heat capacity of the reinforcement system over the determined temperature range, 20 (RT)–220 °C.

(11) $$C_{p_{c}}=m_{f}C_{p_{f}}+m_{r}C_{p_{r}}$$

where $C_{p_{c}}$, $C_{p_{f}}$, and $C_{p_{r}}$ represent the specific heat capacities of the composite, fiber, and resin, respectively.

**Figure 10:**
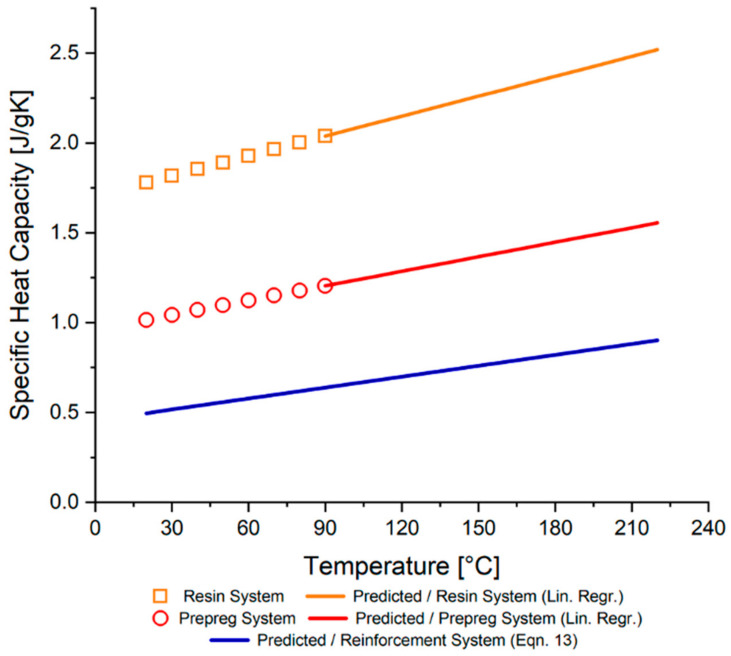
Experimental and predicted specific heat capacity values of the constituent materials.

Figure 10 depicts the specific heat capacity values of the prepreg system constituents, predicted using a rule of mixtures model for specific heat capacity and linear regression. The results reveal that the specific heat capacities of the constituents exhibit a noticeable increase following a linear trend.

#### 3.3.2. Thermal Conductivity

**Figure 11:**
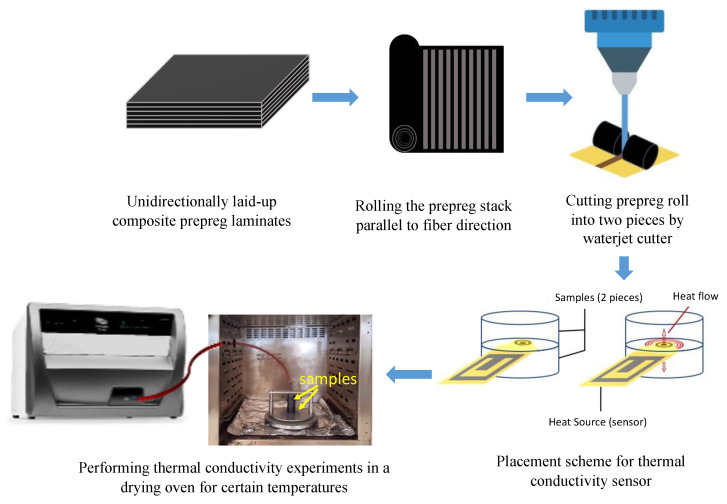
Experimental procedure of thermal conductivity characterization for the prepreg system.

The thermal conductivity of the prepreg system and the resin itself were characterized in different temperatures to obtain the intrinsic thermal conductivities of the components. In this study, a novel experimental strategy towards sample preparation was developed to characterize the thermal conductivity of the prepreg system and its constituents. Figure 11 represents an overview of the experimental strategy measurements that were carried out with a thermal constant analyzer (Hot Disk, TPS 2500S model, Göteborg, Sweden) in an oven to maintain the determined temperature of the experiment medium, as previously demonstrated in the literature [36]. To measure the thermal conductivity of the prepreg system, the five prepreg plies (300 × 300 mm^2^) were rolled in parallel to the unidirectionally aligned fibers to form a cylinder. Next, the cylinder was cut into two identical pieces by a water jet cutter to avoid distracting the fiber orientations. Subsequently, two cylindrical samples with the dimensions of 50 mm diameter and 50 mm height were obtained. A temperature sensor was placed on top of the fist cylinder and the second one was placed above the first one. The imbricated samples were installed into a pressing mechanism to avoid the gap between the sensor and samples. In this method, major assumptions are that the unidirectional (UD) fibers are perfectly aligned in-line and that distractions within the fiber orientations were successfully avoided, which means that the UD fibers of the two samples are perfectly matched during the test. Later, the test samples were placed in a drying oven to conduct the experiments in a temperature-controlled environment. Since the prepreg system used in this study comprises a fast cure resin with a cure cycle between 80 and 130 °C, two sets of experiments were performed prior to the cure cycle at 25 and 60 °C.

For the thermal conductivity characterization of the resin system, the epoxy resin was removed from the freezer and kept at room temperature to allow the resin to liquify. Later, a small portion of resin was loaded upon the flat surface of the sensor with a diameter of 50 mm and a height of at least 10 mm. Subsequently, the sensor was placed in a drying oven to maintain the predetermined temperature of the experiment medium. With this experiment, a single thermal conductivity value of the resin system was obtained, representing both in-plane and through-thickness directions due to the isotropy. Experiments were performed at 25 and 60 °C for the resin system as well.

Anisotropy of the prepreg system leads to different thermal gradients in accordance with the heat flow through different fiber directions [36]. For unidirectional composites, the commonly adopted thermal conductivity models were developed by Springer and Tsai for the in-plane, k_||_, (along the fibers, in-plane) and transverse, k_⊥_, (perpendicular to the fibers, through-thickness) directions as described below [37].

(12) $$k_{\left|\right|}=v_{r}k_{r}+v_{f}k_{f}$$

(13) $$k_{⊥}=\frac{1}{\frac{v_{f}}{k_{f}}+\frac{v_{r}}{k_{r}}}$$

where k_r_ and k_f_, v_r_, and v_f_ are the thermal conductivities and volume fractions of the resin and fiber, respectively. Anisotropic thermal conductivity (in-plane and through-thickness) for the prepreg system and isotropic thermal conductivity values for the matrix (resin) material were obtained from the experiments performed at 25 and 60 °C. 

Table 7 summarizes the anisotropic thermal conductivity results in the in-plane and through-thickness directions for the prepreg samples and isotropic thermal conductivity results for the resin material obtained from the experiments performed at 25 and 60 °C. Comparing the findings with those of other studies confirms that the thermal conductivity values do not demonstrate a noteworthy change with the temperature [18,20,37]. Therefore, the values obtained through these experiments can be considered the reference values for the prepreg system thermal conductivity. Despite the scarcity of data points for the thermal conductivity behavior, this tendency substantiated previous findings in the literature [20].

## 4. Conclusions

In this study, a comprehensive methodology that systematically characterizes material properties and the constitutive behavior of a vacuum bag only prepreg system was developed to develop an integrated Vacuum Bag Only process model and to invigorate its accuracy. This approach was established based on three primary process parameters: heat transfer, flow through porous media, and consolidation.

First, the cure-dependent properties of the epoxy resin were characterized to predict the cure kinetics, rheology, and glass transition temperature behavior under Vacuum Bag Only cure conditions. The cure-dependent model parameters were obtained by adopting the least-squares methods to fit the experimental data determined through Dynamic Scanning Calorimetry, Rheometer, and Dynamic Mechanical Analysis, respectively. For this, a diffusion-controlled cure kinetics model accurately predicted the cure kinetics behavior of the epoxy resin and exhibited excellent agreement with the dynamic and isothermal Dynamic Scanning Calorimetry experiments. Subsequently, a phenomenological viscosity model was applied to successfully estimate the resin rheological behavior as a function of temperature and degree of cure. The model demonstrated a reasonable agreement with the rheology experiments at different dynamic conditions. Lastly, the DiBenedetto equation was employed to describe the glass transition temperature evolution of the epoxy resin system with the degree of cure.

Second, first fiber architecture was investigated as the main microstructural evolution is the resin flow into dry regions. The resulting micrographs exhibited that the domain wherein resin flow and air evacuation occur consisted of elliptical dry fiber tow areas, containing randomly packed fibers, surrounded by resin-rich regions. This was followed by void-content and the fiber volume fraction analyses of the prepreg system through sets of x-ray tomography scans of the laminates processed to different curing stages. Dry fiber tow areas were initially significantly increased after the consolidation and air evacuation and stabilized after the resin reached. Furthermore, microscopic transverse permeability of the fabric was calculated through a numerical analysis for a micro-scale domain consisting of random packs of fibers and resin-rich regions. This predicted permeability value then can be inputted as an initial permeability value for the subsequent mathematical modeling and numerical analysis.

Third, specific heat capacity and thermal conductivity of the prepreg system constituents were characterized to better describe the heat transfer of the laminates during the Vacuum Bag Only prepreg processing and to include them into the integrated process model to be established. A series of experiments were performed through Dynamic Scanning Calorimetry and Thermal Constant Analysis to predict the evolution of specific heat capacity and thermal conductivity of the prepreg system with the temperature, respectively. Hence, the evolution of these two fundamental thermal properties and their effects on the heat transfer of the system were able to be characterized and incorporated into the subsequent process modeling.

Conclusively, the devised approach can be accepted as a starting point towards establishing a process design methodology that integrates characterization, modeling, optimization, and verification to produce high-performance composite structures through Out-of-Autoclave techniques with the allowed void content. In future work, the results of this study will be used as an input to develop integrated Vacuum Bag Only prepreg process modeling.

## Figures and Tables

**Table 1:** OM12 resin system cure kinetics model parameters.

Model Parameters	Units	Value
Heat of Reaction (∆H)	J/g	340
Pre-exponential cure rate coefficient of reaction I (A_1_)	$s^{-1}$	$1.410\times 10^{5}$
Activation energy of reaction I (E_A1_)	J/mol	$66.190\times 10^{3}$
Pre-exponential cure rate coefficient of reaction II (A_2_)	$s^{-1}$	$1.320\times 10^{2}$
Activation energy of reaction II (E_A2_)	J/mol	$89.505\times 10^{3}$
First exponential constant (m_1_)	-	0.708
Second exponential constant (m_2_)	-	0.901
Third exponential constant (n_1_)	-	1.754
Fourth exponential constant (n_2_)	-	0.500
Diffusion Constant (D)	-	88.970
Critical degree of cure at absolute zero temperature (α_C0_)	-	−0.669
Increase in critical resin degree of cure with temperature (α_CT_)	${}^{{}^{\circ}}K^{-1}$	$7\times 10^{-4}$

**Table 2:** Glass transition temperature model parameter.

Final Parameters	Units	Value
$T_{g_{0}}$	°C	−1.6900
$T_{g\infty }$	°C	139.5400
λ	-	0.6221

**Table 3:** OM12 resin system rheology model parameters.

Model Parameters	Units	Value
Pre-exponential viscosity coefficient I ($A_{\mu_{1}}$)	$s^{-1}$	$2.49\times 10^{-19}$
Activation energy of reaction I ($E_{\mu_{1}}$)	J/mol	$91\times 10^{3}$
Pre-exponential viscosity coefficient II ($A_{\mu_{2}}$)	$s^{-1}$	$3.4\times 10^{-8}$
Activation energy of reaction II ($E_{\mu_{2}}$)	J/mol	$46\times 10^{3}$
First exponential constant (A)	-	10.00
Second exponential constant (B)	-	−15.00
Third exponential constant (C)	-	1.30
Degree of cure at gel point ($\alpha_{\mathrm{gel}}$)	-	0.75

**Table 4:** Micro-CT scanning parameters.

Parameter	Unit	Value
Filter	-	None
X-ray voltage	kV	62
X-ray intensity	µA	161
Resolution	µm/pixel	1.75–3
Image size	pixels	4000 × 2096

**Table 5:** The evolution of void content, average ply thickness, and fiber volume fraction for each sample.

Parameters\Samples	Uncured/1-Layer	Cured/1-Layer
Void content (%)	17.60	0.88
Average ply thickness (mm)	0.5 (0.125 for each resin film and 0.25 for dry fiber bed)	0.30
Fiber volume fraction (%)	33.5 (micro-CT) 31.4 (Soxhlet)	67.59

**Table 6:** Modeling parameters for the numerical permeability analysis.

Parameter	Value
Viscosity, μ	0.1 Pa·s
Fiber orientation	Hexagonal packing
Fiber diameter	3 μm
Unit cell dimensions	0.05 mm×0.05 mm
Cylinder boundary conditions	No-slip (u=0)
Outer boundary conditions (left–right)	Slip (u·n=0)
Pressure drop (top–bottom)	1000 Pa

**Table 7:** Anisotropic/isotropic thermal conductivity results of the experiments.

Samples/Parameters	Ambient Temperature (°C)	$k_{\|}$ (W/mK)	$k_{⊥}$ (W/mK)
KOM12 Prepreg System	25	5.5444	0.44439
	60	6.5199	0.41319
OM12 Epoxy Resin System	25	0.1934	
	60	0.1549	

## Data Availability

The data presented in this study are contained within the article.
